# Breaking Through Cancer Pain: From Single‐Drug Management to Multimodal Analgesia

**DOI:** 10.1155/prm/5161889

**Published:** 2026-07-31

**Authors:** Yating Xu, Shu Xu, Bo Huang, Zefeng Wang, He Liu, Jing Zhuang

**Affiliations:** ^1^ Huzhou Central Hospital, Fifth School of Clinical Medicine of Zhejiang Chinese, Medical University, Huzhou, Zhejiang, China, gumed.edu.pl; ^2^ Huzhou Central Hospital, Affiliated Central Hospital, Huzhou Normal University, Huzhou, Zhejiang, China, hzhospital.com; ^3^ Zhejiang-France United Laboratory of Integrated Traditional Chinese and Modern Medicine in Colorectal Cancer, Huzhou, Zhejiang, China; ^4^ College of Information Engineering, Huzhou Normal University, Huzhou, Zhejiang, China

**Keywords:** cancer pain, integrative therapies, intrathecal drug delivery, nanomedicine, neuromodulation

## Abstract

**Background:**

Cancer pain affects 60%–80% of patients with advanced cancer, and approximately 30% of patients experience inadequate pain control. Although the World Health Organization (WHO) three‐step analgesic ladder has substantially improved pain management, inadequate analgesia, opioid‐related adverse effects, and refractory pain continue to pose significant clinical challenges.

**Objective:**

This narrative review critically evaluates recent advances in cancer pain management, including novel analgesics, optimized opioid formulations, nanomedicine, invasive interventions (intrathecal drug delivery, neuromodulation, and neurolysis), and complementary integrative therapies, aiming to provide up‐to‐date insights for clinicians and researchers.

**Results:**

Novel analgesics targeting μ‐opioid receptor bias, ion channels, and multitarget strategies have shown preclinical promise. Improved opioid formulations and nanomedicine‐based approaches may enhance drug delivery and reduce toxicity. Fourth‐step interventions, including intrathecal drug delivery, neuromodulation, and neurolysis, provide targeted options for selected patients with refractory cancer pain. Integrative therapies, including mind–body interventions, acupuncture, massage, music therapy, and game‐based approaches, may improve symptom burden and quality of life, although evidence for direct analgesic effects remains limited.

**Conclusion:**

Cancer pain management is evolving toward a multimodal and increasingly personalized framework. Despite substantial progress, many emerging therapies lack robust cancer‐specific validation. High‐quality clinical trials, standardized treatment protocols, and improved translational strategies are needed to establish evidence‐based precision cancer pain management.

## 1. Introduction

Cancer pain is a common complication in patients with advanced cancer, caused by tumor burden, metastasis, antitumor therapies, and psychological factors [1]. It markedly impairs quality of life and hinders cancer treatment. The prevalence reaches 60%–80% in advanced stages, with about one‐third suffering severe pain [2]. Cancer pain includes nociceptive, neuropathic, and mixed types, driven by interactions among immune, nerve, and cancer cells in the tumor microenvironment [3]. Its heterogeneity is affected by tumor features, treatment side effects, and individual genetic, psychological, and social factors, posing major clinical challenges. Approximately 30% of elderly patients experience uncontrolled pain, and the overall inadequate analgesia rate is up to 70% [4]. The high prevalence, complexity, and poor control of cancer pain make it a critical issue in oncology.

For a long time, cancer pain management has been based on the World Health Organization (WHO) three‐step analgesic ladder, centered on nonsteroidal anti‐inflammatory drugs (NSAIDs), weak/strong opioids, and adjuvant analgesics [5]. However, its effectiveness remains limited by opioid‐related adverse effects and dependence, inadequate control of neuropathic pain, and the restricted applicability of interventional therapies [6–8].

Recent advances have shifted cancer pain care from single‐modality pharmacotherapy to multidimensional integrated management, encompassing optimized pharmacotherapies, “fourth‐step therapy” for refractory pain, and complementary adjuvant therapies, establishing a patient‐centered framework. Conventional pharmacotherapy remains fundamental but is increasingly insufficient. Future research should focus on personalized strategies and wider clinical implementation. By clarifying the role of each intervention in the multimodal framework, this narrative review aims to provide up‐to‐date insights for clinicians and researchers, ultimately improving pain control and quality of life for cancer patients.

## 2. Methodology

This narrative review, based on a critical evaluation of the literature on cancer pain, has summarized and organized the existing research findings on cancer pain management. Electronic searches were conducted in PubMed, Web of Science, and Embase databases to identify literature from 1985 to the present. The following search terms were used in various combinations across all databases: “cancer pain,” “multimodal analgesia,” “opioids,” “intrathecal drug delivery,” “neuromodulation,” “neurolysis,” and “integrative therapies.” Titles and abstracts were initially screened, followed by full‐text review to verify eligibility for cancer pain‐related studies. Inclusion criteria comprised clinical trials, guidelines, case reports, and systematic reviews directly addressing recent advances in cancer pain management. Exclusion criteria included articles not published in English, conference abstracts, and studies unrelated to the topic of cancer pain management. A table summarizing the levels of evidence of the studies cited in this review has been provided in the appendix.

### 2.1. Drug Therapy

**FIGURE 1:**
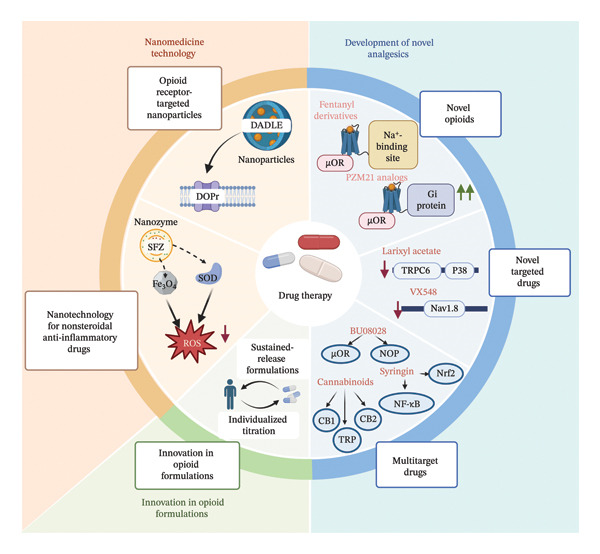
Schematic diagram of innovative pathways in pharmacotherapy for cancer pain. This figure illustrates three core innovative directions in pharmacotherapy for cancer pain, highlighting key strategies to improve analgesic efficacy, safety, and targeting through novel analgesic development, optimized opioid formulations, and nanomedicine‐based targeted delivery.

The WHO three‐step analgesic ladder remains the foundation of cancer pain management and guides pharmacological selection according to pain intensity [9]: Weak opioids are combined with nonopioid analgesics or NSAIDs, whereas strong opioids are used for moderate‐to‐severe pain. Treatment typically begins with low doses and is adjusted through titration to balance analgesia and adverse effects. However, opioid tolerance and dependence may reduce long‐term efficacy [10], shifting research focus from conventional pain relief toward more precise management of refractory pain. As illustrated in Figure 1, current pharmacological innovations focus on three areas: novel analgesics, improved opioid formulations, and nanomedicine‐based approaches.

#### 2.1.1. Development of Novel Analgesic Agents

Current analgesic drug development mainly focuses on three directions: optimizing opioid molecular structures to reduce respiratory depression and addiction risks, targeting ion channels and adopting multitarget synergistic strategies to avoid drug resistance.

Conventional μ‐opioid receptor (μOR) agonists provide effective analgesia but are limited by systemic toxicity. To improve safety, preclinical studies have designed bivalent fentanyl derivatives that target both μOR and the conserved sodium ion‐binding site of Class A G protein–coupled receptors (GPCRs) [11]. In addition, G protein–biased μOR activation is associated with fewer adverse effects. Optimized PZM21 analogs, tested only in animal models, show enhanced G protein signaling bias and higher lipophilicity, improving central nervous system (CNS) penetration [12]. Although these agents have shown promising efficacy in noncancer pain models, whether these findings can be reproduced in cancer‐associated pain remains unclear.

The transient receptor potential (TRP) superfamily comprises multiple subfamilies involved in pain signaling. Recent evidence identifies TRPM3 as a novel target for alleviating oxaliplatin‐induced peripheral neuropathic pain, as genetic or pharmacological inhibition of TRPM3 effectively reduces mechanical allodynia and cold hypersensitivity in preclinical models [13]. Meanwhile, intrathecal larixyl acetate (LA) inhibits TRPC6 and p38 signaling in microglia, producing analgesic and anti‐inflammatory effects in preclinical models of neuropathic pain [14], suggesting potential for refractory chronic pain. The voltage‐gated sodium channel subtype Nav1.8 is another key target, as it is highly and selectively expressed in primary nociceptive neurons. Two Nav1.8 inhibitors, VX150 and VX548, have demonstrated significant analgesic efficacy in clinical trials for acute and noncancer neuropathic pain [15]. Suzetrigine (VX548), the first FDA‐approved Nav1.8 inhibitor, represents a novel nonopioid analgesic [16]. However, its efficacy in cancer pain remains limited and uncertain, as no dedicated study in cancer patients has been conducted to date.

Multitarget drug strategies may improve analgesic efficacy while reducing adverse effects and drug resistance [17]. In preclinical studies, BU08028, a dual μOR/NOP receptor ligand, enhances analgesia while reducing opioid abuse liability [18]. Cannabinoids, which target CB1/CB2 receptors, TRP channels, and inflammatory pathways, have shown potential benefits for cancer‐related pain and anxiety in a recent systematic review and meta‐analysis [19]. Emerging immunotherapeutic approaches targeting PD‐1/PD‐L1 and STING remain at the preclinical stage. Specifically, PD‐1/PD‐L1 signaling regulates bone cancer pain via the JNK/CCL2/CCR2 axis [20], whereas the STING agonist DMXAA exerts antitumor, bone‐protective, and analgesic effects through Type I interferon signaling, suggesting potential to overcome limitations of single‐target therapies [21].

#### 2.1.2. Advancements in Opioid Formulation Development

Opioids, such as morphine and fentanyl, constitute the cornerstone of pharmacological management for moderate‐to‐severe cancer pain. Although effective, long‐term use is associated with adverse effects, including gastrointestinal symptoms, sedation, and respiratory depression, which may reduce patient tolerance and adherence [10]. Opioid tolerance or dependence may further diminish analgesic efficacy. Accordingly, clinical management increasingly emphasizes individualized dose titration to balance efficacy and adverse effects.

Careful individualized dose titration remains fundamental to optimizing opioid therapy, with sustained‐release formulations generally preferred in clinical practice. By providing continuous analgesia, reducing dosing frequency, and minimizing fluctuations in plasma drug concentrations, these formulations can improve both treatment adherence and tolerability. A retrospective clinical study reported that high‐dose controlled‐release oxycodone effectively relieved moderate‐to‐severe cancer pain with manageable adverse effects [22]. Similarly, a case report described successful pain control in a patient with severe bone metastasis pain secondary to small‐cell lung cancer using high‐dose oxycodone beyond conventional guideline recommendations [23]. Although these findings highlight the potential value of individualized dosing strategies, both studies represent low‐level evidence, and the optimal opioid dosing regimen remains undefined. Therefore, dose escalation should be guided by careful assessment of analgesic benefit and adverse effects, with close monitoring of cumulative toxicity rather than pursuing complete pain relief alone.

Although individualized titration and sustained‐release formulations may optimize opioid therapy, evidence supporting off‐guideline dosing remains limited. Further high‐quality studies are needed to establish evidence‐based dosing strategies for personalized cancer pain management.

#### 2.1.3. Nanomedicine Technology

Opioid receptors, including δ, μ, and κ types (DOPr, MOPr, and KOPr), mediate analgesia by suppressing neuronal excitability. Based on this mechanism, preclinical studies have developed nanoparticles targeting nociceptor endosomes: The δ‐opioid receptor agonist [D‐Ala2, D‐Leu5 enkephalin] (DADLE) was encapsulated into lipid‐coated mesoporous silica core–shell nanoparticles. These nanoparticles bind to membrane DOPr, are internalized into endosomes of dorsal root ganglion cells, and release DADLE under acidic, reductive conditions, achieving prolonged anti‐inflammatory and analgesic effects in animal models [24]. However, this strategy’s translation is currently limited by the lack of disease‐specific efficacy data.

NSAIDs are widely used for chronic pain management, primarily acting by inhibiting cyclooxygenase (COX) to reduce prostaglandin synthesis. However, they cannot eliminate pain‐related pathological factors such as reactive oxygen species (ROS) and protons (H^+^), and nanotechnology has opened new avenues to enhance their efficacy [25]. For instance, the antioxidative nanozyme SFZ (SOD&Fe_3_O_4_@ZIF‐8), when intrathecally administered in animal models, degrades in the acidic inflamed tissue microenvironment, releasing superoxide dismutase (SOD) and Fe_3_O_4_ nanoparticles. These synergistically scavenge ROS, reduce inflammatory cytokines, and alleviate pain [26]. To address NSAIDs’ limitations in severe pain, the multifunctional nanocomposite LDH/AZ‐ALD targets bone tumor sites, where it neutralizes H^+^, promotes bone repair via Mg^2+^ release, and suppresses TrkA‐mediated aberrant nerve hyperplasia, achieving triple effects in murine models [27]. Their potential clinical benefits remain speculative until validated in cancer‐specific models and human trials.

Nanomedicine has shown promise in preclinical studies for addressing limitations of conventional pain therapies, such as poor targeting, low bioavailability, and off‐target toxicity. Nanocarriers can cross biological barriers and enable multimodal combinations in animal models. However, clinical translation remains hindered by challenges in manufacturing consistency, limited long‐term safety data regarding biodistribution and tissue accumulation, and the absence of dedicated human trials in cancer pain. Future research should prioritize standardized manufacturing processes, comprehensive safety evaluation, and well‐designed clinical studies.

### 2.2. Invasive Interventions for Cancer‐Related Pain

The WHO three‐step analgesic ladder remains the cornerstone of cancer pain management and has greatly improved pain control worldwide. However, 10%–20% of patients do not achieve adequate analgesia with strong opioids [28], particularly in the presence of bone metastasis or nerve invasion. Minimally invasive interventions have traditionally been reserved for refractory pain or end‐of‐life care, and delayed implementation may prolong severe pain and adversely affect quality of life and adherence to anticancer treatment.

**FIGURE 2:**
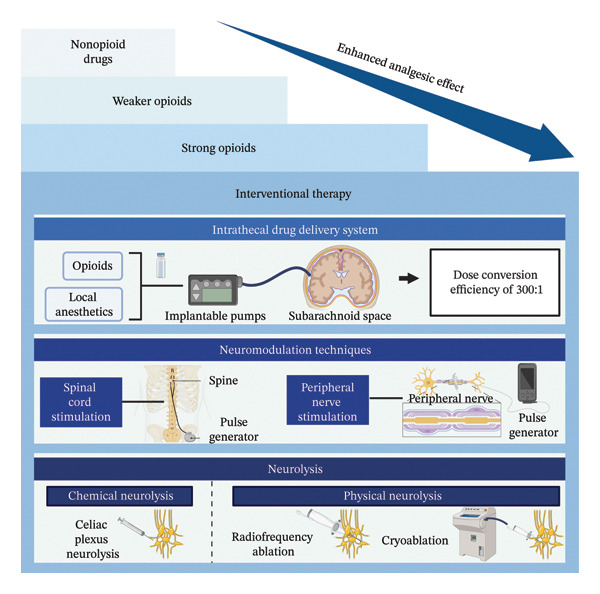
Schematic diagram of core interventional techniques for cancer pain. This figure presents the main interventional approaches of the fourth‐step therapy for refractory cancer pain, including intrathecal drug delivery, neuromodulation, and neurolysis, illustrating their mechanisms for effective and minimally toxic analgesia.

To address this gap, the concept of fourth‐step therapy has been proposed, emphasizing earlier consideration of interventional techniques when standard analgesia is insufficient, adverse effects are intolerable, or a definable pain generator is present. As summarized in Figure 2, these core techniques include targeted intrathecal drug delivery via an implanted pump, neuromodulation via spinal cord or peripheral nerve stimulation (PNS), and neurolytic interventions such as chemical or radiofrequency ablation (RFA) of specific nerve plexuses. When applied appropriately, fourth‐step therapy may improve pain control, reduce systemic opioid exposure, and enhance quality of life. However, its use requires careful patient selection, technical expertise, and multidisciplinary collaboration; it is not intended to replace the WHO ladder but to complement it for refractory cases.

#### 2.2.1. Intrathecal Drug Delivery System (IDDS)

Epidural and peripheral nerve catheters provide effective short‐term analgesia, whereas IDDSs are preferred for refractory cancer pain due to improved efficacy and reduced systemic toxicity [29]. IDDS delivers microdoses directly to the spinal dorsal horn, achieving a dose conversion ratio of up to 300:1 and significantly reducing systemic opioid exposure [30]. The 2017 Multimodal Analgesia Consensus recommends IDDS for patients receiving ≥ 200 mg/day oral morphine equivalent (OME), inadequate pain control, or intolerable systemic side effects [31], which is also commonly used as a pragmatic threshold for early consideration.

Clinical guidelines list morphine and hydromorphone as first‐line intrathecal drugs. However, 15%–20% of patients still have poor analgesia or side effects with intrathecal morphine. A controlled noninferiority trial has demonstrated that hydromorphone may serve as a viable alternative owing to its higher potency and superior tolerability [32]. Adding intrathecal bupivacaine to opioids has been shown in a retrospective cohort study to reduce median daily OME from 503 mg preimplant to 105 mg postimplant [33].

As a fourth‐step intervention for refractory cancer pain, IDDSs provide targeted analgesia for patients unresponsive to optimized systemic pharmacotherapy. Their use is associated with significant risks, including infection, bleeding, catheter‐related malfunction, pump failure, and respiratory depression, requiring strict patient selection within a multidisciplinary framework. Despite these limitations, established practice data indicate that IDDS can reduce opioid consumption and prolong home‐based palliative care in advanced cancer patients [34].

#### 2.2.2. Neuromodulation Techniques

Neuromodulation represents a fourth‐step intervention for carefully selected patients with refractory neuropathic cancer pain, particularly those who have failed pharmacological and interventional treatments. Patient selection typically requires confirmed neuropathic pain etiology, inadequate response to conventional therapies, acceptable psychological status, and sufficient cognitive ability to participate in device management. However, its clinical use is constrained by limited high‐quality evidence and restricted accessibility in specialized centers.

Spinal cord stimulation (SCS) is an established modality for chronic neuropathic pain, including complex regional pain syndrome and refractory spinal pain. Epidural electrode implantation modulates dorsal column signaling and central sensitization, thereby reducing pain perception. Recently, paresthesia‐free SCS has been introduced, providing analgesia without paresthesia and improving comfort over traditional low‐frequency SCS [35]. Its exact mechanism remains unclear and may differ from conventional SCS; possible pathways include reduced central sensitization and decreased neuronal hyperpolarization in the superficial dorsal horn [36]. In cancer pain, however, evidence is limited to small observational studies, with no large randomized controlled trials. Therefore, SCS remains investigational in oncology and should be considered only in highly selected patients after failure of standard therapies.

PNS is a less invasive neuromodulation technique, indicated when SCS is contraindicated or pain is localized to a peripheral nerve territory. It involves percutaneously implanting a thin electrode lead near the target peripheral nerve, connected to a small implantable pulse generator. PNS modulates peripheral nerve activity to affect central neuronal circuits, similar to acupuncture and TENS [37]. In cancer pain, the evidence for PNS is extremely limited. A case series of 12 refractory oncological pain patients (7 receiving PNS) showed mean pain scores dropped from 9.0 (SD 1.0) to 3.1 (SD 1.6) 60 days postimplant, with analgesia persisting months after device removal [38]. For PNS, current evidence is derived primarily from small retrospective case series reporting analgesic benefit, but the absence of randomized controlled trials and potential selection bias limits its interpretability in oncology populations. Consequently, PNS should be regarded as an experimental technique in cancer pain management.

Overall, both SCS and PNS are implant‐based techniques with potential benefit in carefully selected patients, but remain limited to specialized centers due to evidence gaps and procedural risks.

#### 2.2.3. Neurolysis

Neurolysis is a procedure to manage intractable pain by destroying specific nerves via chemical agents, RFA, cryoablation, or neurosurgery, mainly classified into chemical (e.g., celiac plexus neurolysis, CPN) and physical (RFA, cryoablation) types. Patient selection typically includes well‐localized visceral pain and failure of systemic analgesics.

A retrospective cohort study suggests early neurolysis yields better therapeutic effects [39], so it is recommended to incorporate it into initial treatment rather than as a salvage option [40]. Image guidance (fluoroscopy, ultrasound, CT, or EUS) is essential for safety and efficacy; EUS reduces procedural complexity and neurological complications [41]. RFA can be an alternative for pancreatic cancer patients unresponsive to CPN, with analgesia lasting 3–12 months [42]. Cryoablation, using extreme cold to block pain signals, has fewer gastrointestinal side effects than chemical neurolysis (e.g., ethanol) [43]. Overall, physical ablation (cryoablation, RFA) is superior to chemical neurolysis with fewer adverse effects and more predictable efficacy.

Despite guideline recommendations, neurolysis remains underutilized due to limited access to trained interventionalists and imaging resources, as well as concerns regarding irreversible complications.

### 2.3. Complementary and Integrative Therapies

**FIGURE 3:**
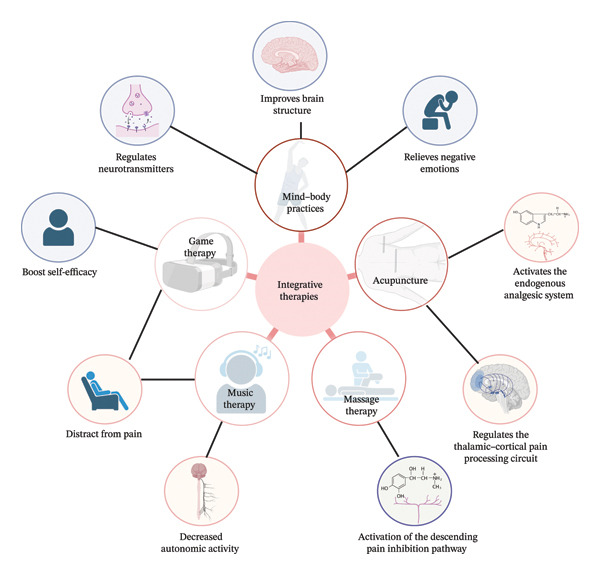
Mechanisms of action of integrative therapies for cancer pain. This figure summarizes the main mechanisms by which integrative therapies relieve cancer pain, including mind–body practices, acupuncture, music therapy, massage, and game‐based interventions. It highlights their actions on physical, psychological, and neural pathways to complement conventional analgesic treatments.

Pharmacotherapy and invasive interventions remain the mainstay of cancer pain management. Nonpharmacological integrative therapies are increasingly used as supportive measures. As shown in Figure 3, these therapies exert effects through mechanisms like neurotransmitter modulation, attention diversion, mood improvement, and endogenous analgesic system activation. However, current evidence for their specific analgesic effects in cancer pain is limited.

#### 2.3.1. Mind–Body Interventions

Mind–body practices focus on the interaction among the brain, mind, body, and behavior, aiming to improve physical function and overall health via cognitive regulation. These include meditation, yoga, and tai chi, which may alleviate negative emotions and improve physical and psychological well‐being.

A meta‐analysis of yoga in breast cancer patients reported reduced pain intensity (SMD: −0.38, 95% CI −0.74 to −0.02) and improved physical and mental health and sleep [44]. Meanwhile, a separate study in healthy volunteers found that experienced yoga practitioners had higher cold pain tolerance, which was associated with insula gray matter volume and practice duration, but this study did not include cancer patients, limiting its applicability to cancer pain [45]. Furthermore, a systematic review identified that only 3 out of 690 studies validated the proposed “yoga–interoception–pain relief” pathway, highlighting the lack of mechanistic evidence [46].

Multiple systematic reviews show varying effects of mind–body practices in cancer patients. Baduanjin reduces moderate‐to‐severe cancer‐related fatigue (OR = 0.27, 95% CI 0.17–0.42) [47]. A pooled analysis of 15 studies [48] found mind–body exercises improved physical fitness (SMD = 0.46), sleep (MD = −0.66), and depression (SMD = −0.21). Results for Tai Chi Chuan (TCC) are inconsistent: One meta‐analysis [49] of 322 breast cancer patients showed only upper limb strength improvement, while another trial [50] of 885 patients reported reduced pain (SMD = 0.30) and better shoulder function and anxiety after 12 weeks of TCC. Such variability may reflect differences in patient populations, intervention protocols, and study methodology.

Mind–body interventions appear beneficial for psychological well‐being, sleep, fatigue, and physical function in cancer patients, whereas evidence supporting a direct analgesic effect remains inconsistent.

#### 2.3.2. Acupuncture

Acupuncture, a traditional therapeutic approach rooted in Traditional Chinese Medicine (TCM), boasts a long and well‐documented history [51]. It is widely used in clinical settings and has been recognized for its advantages in alleviating pain, fatigue, and nausea. It is generally considered cost‐effective, accessible, and adaptable for pain management [52], with various modalities including single‐needle acupuncture, warm needle acupuncture, electroacupuncture, and bee venom acupuncture. Additionally, it can be combined with Western analgesics, Chinese herbal medicine, or acupoint injections [53]. Based on available clinical evidence, it was included in ASCO’s 2016 clinical practice guidelines as an adjunctive option for cancer‐related pain management [54].

Proposed mechanisms by which acupuncture may alleviate pain include regulation of neurotransmitters (endorphins, endogenous opioid peptides, serotonin) and modulation of brain region functions [55, 56]. However, most of these mechanistic insights come from preclinical animal studies or small exploratory human neuroimaging studies. For example, electroacupuncture activates μOR and δ‐opioid receptors and enhances enkephalin release in the rat spinal dorsal horn, whereas wrist‐ankle acupuncture reduced spinal 5‐HT3 receptor expression in a mouse neuropathic pain model, resulting in a 31.2% reduction in receptor‐positive cells [57]. fMRI studies in healthy volunteers or small patient samples suggest that acupuncture modulates thalamocortical circuits and default mode network activity [58].

In a randomized controlled trial, 7‐day transcutaneous electrical acupoint stimulation (TEAS) significantly reduced NRS pain scores by 1.75 points and relieved opioid‐induced gastrointestinal adverse reactions in patients with hepatocellular carcinoma (HCC) [59]. A meta‐analysis of 17 RCTs including 1162 patients reported that acupuncture, alone or combined with other medications, significantly reduced cancer‐related pain (*p* < 0.05) [60]. The analgesic effect was strongest when combined with Western medicine for ≤ 14 days (RR = 1.69, 95% CI 1.42–1.99), and younger patients showed better responses than older ones. The included RCTs exhibit high heterogeneity in cancer types, acupuncture modalities, control interventions, and outcome measures.

Acupuncture is a widely used adjunctive therapy that may improve cancer‐related symptoms and enhance multimodal pain management. However, substantial heterogeneity in study design and intervention protocols limits the certainty of current evidence.

#### 2.3.3. Massage Therapy

Massage therapy is commonly used as an adjunctive intervention for cancer pain and may exert its effects through the activation of descending inhibitory pathways, improved local circulation, and increased release of endorphins and serotonin [61]. However, existing studies are generally limited by small sample sizes and methodological variability, and the influence of cancer type, disease stage, massage technique, and treatment frequency remains unclear [62]. From a Traditional Chinese Medicine perspective, massage is believed to regulate meridian qi and may help alleviate postoperative discomfort in cancer patients [63]. A meta‐analysis of 13 RCTs (*n* = 1000) reported significant reductions in cancer pain following massage therapy [64]: Massage significantly reduced cancer pain (SMD = −1.16, 95% CI −1.39 to −0.93, *p* < 0.00001), with stronger effects in perioperative and hematologic malignancy patients. Sessions of 10–30 min for at least one week showed the best results, with no severe adverse events.

Massage therapy appears safe and may improve patient comfort and symptom burden. However, variability in study design and intervention protocols limits the certainty of current evidence regarding its analgesic effects in cancer pain.

#### 2.3.4. Music Therapy

Music therapy is a clinical intervention delivered by certified therapists, using tailored music listening or creative activities based on patient assessment to promote physical and psychological well‐being [65]. With cross‐cultural historical origins, it has been shown to benefit chronic conditions, such as fibromyalgia and mood disorders [66, 67], and may relieve cancer‐related symptoms [68]. Its proposed mechanisms include reduced autonomic arousal and improved neural synchronization to support neuroplasticity.

An overview of 13 systematic reviews reported that music therapy combined with standard care significantly relieved cancer pain, fatigue, and psychological distress; six reviews that focused on pain consistently found better analgesic effects compared to control conditions [69–71]. However, considerable heterogeneity in intervention protocols and study design limits the certainty of these findings, and the difficulty of blinding may increase susceptibility to expectancy and nonspecific treatment effects.

#### 2.3.5. Game Therapy

Game therapy, based on distraction, self‐efficacy, and mind–body interaction theories, is intended to reduce pain by immersing patients in virtual games to redirect attention from pain. A single quasiexperimental study in pediatric cancer patients (mean age 11.5 years) found that daily 2.3‐h electronic video game (EVG) use reduced morphine consumption (35.9–28.6 μg/kg/day, *p* = 0.003), breakthrough pain scores (7.7–5.4, *p* = 0.001), and 24‐h supplementary analgesic doses (17–9.6, *p* = 0.001), with no adverse effects [72]. However, the study lacked both randomization and a control group, and its findings have not been replicated in adult patients or other cancer populations.

Serious games are therapeutic tools, with customized casual games (first‐person perspective, no visible avatar, single‐player mode, nonimmersive VR) showing the best outcomes [73]. Although customized games outperform commercial off‐the‐shelf (COTS) games clinically, COTS games are more engaging [74]. At present, evidence supporting game therapy for cancer pain remains preliminary, and further research is required to determine how specific game design elements influence clinical outcomes and whether these findings can be generalized across cancer populations [75].

Collectively, integrative therapies may improve psychological well‐being, sleep quality, fatigue, and overall quality of life in patients with cancer. However, evidence supporting their direct analgesic effects remains limited by substantial clinical heterogeneity, methodological variability, and difficulties in blinding, making placebo effects difficult to exclude. Much of the current evidence, particularly for interventions such as acupuncture and mind–body practices, should therefore be regarded as hypothesis‐generating rather than definitive for cancer‐specific populations. At present, these interventions are best considered adjunctive rather than established analgesic therapies and should complement, rather than replace, evidence‐based pharmacological and interventional approaches. Table 1 summarizes the mechanisms, evidence, and clinical characteristics of the major integrative therapies discussed in this review.

**TABLE 1:** Comparison table of integrative therapies for cancer pain.

Therapy type	Core mechanism	Specific forms	Key research data	Application scenarios and precautions
Mind–body practices	Regulation of neurotransmitters (endorphins), improvement of brain structure (insula gray matter increase), alleviation of negative emotions	Yoga, Tai Chi, Baduanjin	Baduanjin reduced moderate‐to‐severe cancer‐related fatigue by 73% (OR = 0.27) [47]. Tai Chi showed SMD = 0.30 for pain relief in breast cancer patients [50].	Suitable for patients needing psychological improvement; individual differences exist, long‐term adherence required.

Acupuncture	Activation of the endogenous analgesic system (endorphins, 5‐HT), modulation of thalamocortical pain processing circuits	Electroacupuncture, warm acupuncture, wrist‐ankle acupuncture	Seven‐day TEAS reduced moderate‐to‐severe HCC pain by 1.75 points on the NRS [59]. Warm acupuncture showed better efficacy than conventional acupuncture, with younger patients responding more effectively [60].	Can be used as an adjuvant to drugs; requires operation by professional physicians; pay attention to aseptic operation to avoid infection.

Massage therapy	Activation of descending pain inhibitory pathways, promotion of hormone release (endorphins, 5‐HT, etc.)	Manual massage, acupoint massage	Meta‐analysis of 13 RCTs showed analgesic SMD = −1.16 [64]. Optimal parameters: 10–30 min/session, lasting ≥ 1 week [62].	Suitable for pain related to muscle tension; avoid direct massage on tumor sites.

Music therapy	Reduction of autonomic nervous system activity, enhancement of neural electrical activity synchrony, distraction from pain	Passive listening, active playing	Six systematic reviews confirmed superior analgesic effects compared to control groups [71]. Combined with standard care, it relieves cancer pain and fatigue [69, 70].	Noninvasive and easy to implement, suitable for patients at all stages; personalized music programs are more effective.

Game therapy	Distraction through immersive experience, reduction of pain perception, enhancement of self‐efficacy	Electronic video games (EVG), customized serious games	Morphine consumption in pediatric patients decreased by 20% [72]. Customized games showed better clinical effects than COTS games [74].	Particularly suitable for children and adolescents; control game duration to avoid addiction.

*Note:* This table provides a systematic comparison of five integrative therapies, focusing on their core mechanisms, specific modalities, key research findings, and areas of application.

### 2.4. Future Perspectives: Toward Precision Cancer Pain Management

The evolution toward multimodal analgesia naturally culminates in the pursuit of precision medicine for cancer pain [76]. Future strategies may eventually incorporate biomarker‐guided selection, leveraging advances in genomics, proteomics, and neuroimaging to identify patient‐specific pain mechanisms and predict treatment responses. For instance, genetic polymorphisms in opioid receptor genes (e.g., OPRM1 [77]), drug‐metabolizing enzymes (e.g., CYP2D6 [78]), and emerging targets like GPR37 or STING could serve as potential biomarkers to guide the choice of analgesics, optimize dosing, and minimize adverse effects [21]. However, most of these candidates remain at the preclinical or early exploratory stage, and none have been validated for routine clinical use in cancer pain.

Similarly, AI and digital health technologies hold theoretical promise for improving cancer pain monitoring and management. Preliminary studies have explored the use of AI algorithms to integrate multimodal data for pain prediction and wearable devices for physiological monitoring [79, 80]. Nevertheless, these approaches are still in early development or proof‐of‐concept phases, with limited validation in real‐world cancer populations. Large‐scale, prospective studies are needed before they can be considered for routine clinical application.

## 3. Conclusion

Cancer pain management is evolving from a predominantly pharmacological approach toward a more multimodal, minimally invasive, and patient‐centered framework. Advances in pharmacotherapy, interventional pain procedures, and integrative therapies have expanded the range of available treatment options and may improve symptom management and quality of life in selected patients. Nevertheless, important evidence gaps remain. Many emerging pharmacological agents and innovative therapeutic strategies are supported primarily by preclinical, exploratory, or heterogeneous evidence and lack robust cancer‐specific validation. Interventional techniques can provide meaningful benefit for carefully selected patients with refractory pain but are constrained by procedural risks, accessibility, and resource requirements. Integrative therapies may improve psychological well‐being, sleep, fatigue, and overall quality of life; however, their specific analgesic effects remain uncertain, and much of the current evidence should be considered hypothesis‐generating.

Overall, multimodal approaches provide a broader framework for individualized cancer pain management and highlight the importance of integrating pharmacological, interventional, and supportive strategies according to patient needs. Future progress will depend on high‐quality clinical trials, improved patient selection, and rigorous evaluation of emerging therapies to ensure that advances in cancer pain management translate into meaningful and sustainable patient benefit.

**Nomenclature**
AI: Artificial intelligence
CI: Confidence interval
CPN: Celiac plexus neurolysis
fMRI: Functional magnetic resonance imaging
GPR37: G protein–coupled receptor 37
IDDS: Intrathecal drug delivery system
μOR: μ‐opioid receptor
Nav: Voltage‐gated sodium channel
NOP: Nociceptin/orphanin FQ peptide receptor
NSAIDs: Nonsteroidal anti‐inflammatory drugs
OME: Oral morphine equivalent
PNS: Peripheral nerve stimulation
RCT: Randomized controlled trial
RFA: Radiofrequency ablation
SCS: Spinal cord stimulation
SMD: Standardized mean difference
TRP: Transient receptor potential
WHO: World Health Organization

## Author Contributions

Conceived and drafted the manuscript: Jing Zhuang, Zefeng Wang, and He Liu.

Wrote the paper: Yating Xu.

Reviewed and sorted out the literature: Jing Zhuang.

Designed and drew figures: Shu Xu and Bo Huang.

## Funding

This study was supported in part by grants from the National Natural Science Foundation of China (NSFC82171227 and NSFC81300957 to He Liu), the Zhejiang Provincial Natural Science Foundation (LY22H090019 to He Liu), the Open Project Fund of the Key Laboratory for NeuroInformation of Ministry of Education (202311KFY00102), the Zhejiang Province Cerebrovascular Disease Diagnosis and Treatment of Traditional Chinese Medicine Multidisciplinary Innovation Team Project, and the “Integration of Medicine, Industry and Information” Collaborative Innovation Center Fund of Yangtze Delta Region.

## Disclosure

All authors read and approved the paper.

## Ethics Statement

The authors have nothing to report.

## Consent

The authors have nothing to report.

## Conflicts of Interest

Dr. He Liu is one member of the Editorial Board in Anesthesiology and Perioperative Science and recuses himself from every editorial procedure of this submission, including peer review and academic decisions. The other authors declare no conflicts of interest.

## Supporting Information

Additional supporting information can be found online in the Supporting Information section.

## Supporting information


**Supporting Information** Appendix 1.docx, the Evidence Quality Assessment Form for Included Studies. Literature types were graded and scored following JBI evidence appraisal criteria to support evidence synthesis in the main manuscript.
