# Supplementary material for: Breaking Through Cancer Pain: From Single‐Drug Management to Multimodal Analgesia
Source: Pain Res Manag. 2026 Jul 31;2026:5161889. doi: 10.1155/prm/5161889 (PMC13426482; doi:10.1155/prm/5161889)
Supplement: Supplementary file 1 — Supporting Information Appendix 1.docx, the Evidence Quality Assessment Form for Included Studies. Literature types were graded and scored following JBI evidence appraisal criteria to support evidence synthesis in the main manuscript. [file PRM-2026-5161889-s001.docx]

Appendix 1: Evidence Quality Assessment Form for Included Studies

| Author | Study design | Population | Indication | Intervention | Efficacy | JBI Level | JBI quality score | level |
| --- | --- | --- | --- | --- | --- | --- | --- | --- |
| Faouzi A et al. 2023  [11] | Experimental study (in vitro + in vivo) | In vitro: µOR-expressing CHO cell lines; In vivo: Adult male C57BL/6J mice (N=5-10 per group, varies by assay) | Pain (thermal, neuropathic, visceral, inflammatory) | C6 guano ICV | Antinociception in all models; G-protein efficacy similar to reference agonists | 5 | 7/9 | Preclinical finding |
| Wang H et al. 2022  [12] | Experimental study (in vitro + cryoEM) | μOR-expressing cell lines (HEK293/CHO) | Pain (μOR agonism) | PZM21 | G protein partial agonist (EC50 3.7–14 nM, Emax 67–89% of morphine); reduced arrestin recruitment vs fentanyl | 5 | 8/9 | Preclinical finding |
| Wang J et al. 2020  [14] | Animal experiment (rat) | Adult male SD rats (220–250 g) | Neuropathic pain | Larixyl acetate | Dose‑dependent relief of mechanical and cold allodynia (ED50 = 13.43 μM) | 5 | 8/9 | Preclinical finding |
| Vaelli P et al. 2024  [15] | In vitro electrophysiology | Human Nav1.8-expressing CHO cell line | Pain (Nav1.8 inhibition) | VX-150m, VX-548 | VX-150m IC50=15 nM; VX-548 IC50=0.27 nM; relief by depolarization | 5 | 8/9 | Preclinical finding |
| Kiguchi N et al. 2019  [18] | Animal experiment (non-human primate) | Adult rhesus monkeys | Acute pain | BU10038 | Potent, long‑lasting antinociception (ED50 = 0.003 mg/kg s.c.) and antihypersensitivity | 5 | 8/9 | Preclinical finding |
| Creangă‑Murariu I et al. 2025  [19] | Systematic review + meta‑analysis | Cancer patients (21,397 from 98 studies) | Cancer‑associated symptoms | Cannabinoids | Pain reduced (MRAW –1.22); anxiety reduced (MRAW –1.30); trends for appetite, CINV, insomnia | 1 | 9/11 | Cancer palliative care |
| Wang K et al. 2020  [20] | Animal experiment (mouse) | Adult C57BL/6 mice | Bone cancer pain (femoral LLC or CMT‑167 inoculation) | Nivolumab or Pdcd1‑/‑ | Reduced bone destruction and alleviated mechanical/thermal pain | 5 | 8/9 | Preclinical finding |
| Wang K et al. 2021  [21] | Animal experiment (mouse) | Adult C57BL/6 mice | Bone cancer pain | DMXAA or ADU‑S100 | Reduced mechanical/cold allodynia, spontaneous pain | 5 | 8/9 | Preclinical finding |
| Wang W et al. 2018  [22] | Retrospective chart review | Advanced cancer patients | Moderate to severe cancer pain | High‑dose controlled‑release oxycodone (>150 mg/d, mean 177.2 mg/d) | Effective rate 90.1% ; mean NRS 7.10 to 2.15 | 4 | 7/10 | Established practice |
| Zhou T et al. 2015  [23] | Case report | 1 patient, 57‑year‑old male, small‑cell lung cancer with bone metastasis | Severe cancer pain (NRS 6–8) | High‑dose OxyContin , max 1800 mg/day | Pain controlled (NRS 2–3) | 5 | 7/8 | Established practice |
| Jimenez‑Vargas NN et al. 2020  [24] | Human biopsy + animal experiment + in vitro | Human colonic biopsies (ulcerative colitis) + mouse DRG neurons, colitis model, CFA inflammatory pain model | Inflammatory pain | DOPr agonists or nanoparticle‑encapsulated DADLE | Sustained increase in rheobase; inhibited colonic nociceptors; reversed CFA‑induced mechanical allodynia for ≥6 h | 5 | 8/9 | Preclinical finding |
| Ling Y et al. 2023  [26] | Animal experiment (mouse) + in vitro | Adult male ICR mice; BV2 microglia, primary microglia/astrocytes | Inflammatory pain | SFZ NPs | Dose‑dependent reversal of mechanical allodynia; inhibited glial activation, MAPK/NF‑κB pathway, and inflammatory cytokines | 5 | 8/9 | Preclinical finding |
| Chu X et al. 2022  [27] | Animal experiment (mouse) | Tumor‑bearing mice | Metastatic bone cancer pain | Alendronate‑decorated LDH nanoshells encapsulating AZ‑23 | Improved pain threshold; enhanced tumor inhibition | 5 | 7/9 | Preclinical finding |
| Ma K et al. 2020  [32] | Multicenter, randomized, single-blind, controlled noninferiority trial | Refractory cancer pain patients (n=233) | Refractory cancer pain | Intrathecal hydromorphone (ITHM) vs. Intrathecal morphine (ITMO) | ITHM noninferior to ITMO in pain relief. ITHM showed advantages in lower dose escalation and breakthrough pain control. | 1 | 9/13 | Established practice |
| Spiegel MA et al. 2020  [33] | Retrospective case series | Cancer patients with IDDS (n=50) | Refractory cancer pain | Intrathecal morphine + bupivacaine | Median OME decreased from 503 to 105; mean VAS 6.6 to 2.7; 84% achieved ≥50% OME reduction at discharge | 4 | 7/10 | Established practice |
| Mitchell A et al. 2023  [34] | Retrospective longitudinal cohort study | Advanced cancer patients with refractory pain (n=45) | Refractory cancer pain | Intrathecal drug delivery system vs comprehensive medical management | IDDS group had significantly more time in community and lower end‑of‑life MEDD | 3 | 9/11 | Established practice |
| Mainkar O et al. 2020  [38] | retrospective case series | Cancer patients with chronic pain refractory to medical/interventional management (n=12) | Oncologic pain | Temporary percutaneous peripheral nerve stimulation | Average pain score decreased from 9.0 to 2.1 during stimulation and 3.1 after extraction | 4 | 7/10 | Investigational therapy |
| Lu F et al. 2023  [39] | Retrospective cohort study | Advanced pancreatic cancer patients with moderate‑to‑severe pain (n=56) | Abdominal/back pain due to pancreatic cancer | CT‑guided celiac plexus neurolysis (early vs delayed) | Early CPN: significantly lower NRS at 3–5 months; lower OME at 2–4 months | 3 | 9/11 | Established practice |
| Behbahani K et al. 2020  [43] | Retrospective cohort study | Intractable abdominal pain patients (n=83) | Intractable abdominal pain | CT-guided cryoablation of celiac plexus vs. Ethanol neurolysis | Similar efficacy, but cryoablation had significantly lower incidence of diarrhea and fewer overall GI side effects | 3 | 8/11 | Established practice |
| Hsueh EJ et al. 2021  [44] | Systematic review & meta-analysis of RCTs | Breast cancer patients (26 RCTs, n=2069) | Breast cancer treatment‑related complications | Yoga intervention | Significant improvements in social, emotional, functional, and physical health, mental health, sleep quality; reduced anxiety, depression, stress, fatigue, and pain | 1 | 10/11 | Investigational therapy |
| Villemure C et al. 2014  [45] | Cross‑sectional observational study | Experienced North American yoga practitioners (n=14) vs matched controls (n=14) | Pain tolerance and brain structure | Long‑term regular yoga practice | Yogis tolerated cold pain >2× longer than controls; greater GM volume in insula, cingulate, S1, S2, MPFC; higher left intransular WM FA | 4 | 7/8 | Investigational therapy |
| Voss S et al. 2023  [46] | Scoping review | Adults with chronic pain (24 studies included) | Chronic pain | Mind‑body practices (yoga, mindfulness, meditation, tai chi, qigong) | Mind‑body practices may improve interoceptive sensibility; only 3 studies directly examined yoga | 5 | 6/11 | Investigational therapy |
| Kuo CC et al. 2021  [47] | Systematic review & meta-analysis of RCTs | Cancer patients (10 RCTs, n=811) | Cancer‑related fatigue, quality of life, sleep quality | Baduanjin Qigong exercise | Reduced moderate‑severe cancer‑related fatigue (OR=0.27, 95% CI [0.17,0.42]); improved QoL (FACT‑B: MD=11.04; EORTC: MD=10.57); improved sleep quality (PSQI: MD=−2.89) | 1 | 9/11 | Investigational therapy |
| Duan L et al. 2020  [48] | Systematic review & meta-analysis of RCTs | Cancer survivors (15 RCTs, n=1461) | Cancer‑related symptoms | Mind‑body exercise (Tai Chi, Qigong, yoga, dance) | Improved physical fitness (SMD=0.46), fatigue (SMD=−0.47), sleep (MD=−0.66), depression (SMD=−0.21), anxiety (SMD=0.27), BMI (MD=1.31); no significant effect on general QoL or stress | 1 | 9/11 | Investigational therapy |
| Pan Y et al. 2015  [49] | Systematic review & meta-analysis of RCTs | Breast cancer patients (9 RCTs, n=322) | Breast cancer treatment‑related side effects | Tai Chi Chuan exercise | Significant improvements in handgrip strength (SMD=0.60), elbow flexion (SMD=0.75), elbow extension (SMD=1.29), abduction (SMD=0.58), horizontal adduction (SMD=0.77); no significant effect on pain, IL‑6, IGF‑1, BMI, physical/social/emotional well‑being, or general QoL | 1 | 9/11 | Investigational therapy |
| Luo XC et al. 2020  [50] | Systematic review & meta-analysis of RCTs | Breast cancer patients (15 RCTs, n=885) | Breast cancer‑related symptoms | Tai Chi Chuan exercise | Significant improvements in QoL (SMD=0.37), pain (SMD=0.30), shoulder function (SMD=1.34), arm strength (SMD=0.44), anxiety (MD=−4.25), fatigue (SMD=−1.11); subgroup: 12 weeks TCC most effective | 1 | 9/11 | Investigational therapy |
| Paice JA et al. 2016  [54] | Clinical practice guideline (Systematic review-based) | Adult cancer survivors | Chronic pain management in cancer survivors | Multimodal approach: non‑pharmacologic and pharmacologic | Multimodal strategy improves function and pain control | 1 | 10/11 | Established practice |
| Zhang C et al. 2020  [57] | Animal experiment (rat) | Female Wistar rats | Cancer‑induced bone pain (Walker 256 cells intra‑tibia) | Wrist‑ankle acupuncture (WAA) vs electroacupuncture (EA) | Both WAA and EA attenuated mechanical hyperalgesia (P<0.05); WAA had faster onset than EA | 5 | 7/9 | Preclinical finding |
| Kong Q et al. 2023  [58] | Randomized crossover trial | Healthy right‑handed adults | Experimental pain | Real acupuncture (SP6, SP9) vs sham acupuncture (non‑penetrating) vs VGAIT (video‑guided acupuncture imagery) vs VGAIT control | Real acupuncture increased all 4 pain thresholds (P<0.05); VGAIT increased leg heat pain (P=0.017) and leg/thumbnail pressure pain (P<0.001) | 2 | 9/13 | Investigational therapy |
| Zhu L et al. 2024  [59] | Randomized controlled trial | Hepatocellular carcinoma patients with moderate‑to‑severe pain (n=104) | HCC‑related moderate‑to‑severe pain | Transcutaneous electrical acupoint stimulation vs sham TEAS | NRS score: 6.5 → 5.0 (P<0.01) maintained at 1‑week follow‑up; BPI‑Q3 and BPI‑Q5 significantly improved (P<0.001); reduced abdominal distension, nausea, vomiting, dry mouth; improved KPS, QOL‑LC, BFI (P<0.05) | 2 | 10/13 | Investigational therapy |
| He Y et al. 2024  [60] | Systematic review & meta-analysis of RCTs | Primary liver cancer patients (17 RCTs, n=1162) | Primary liver cancer (PLC)‑related pain | Acupuncture | Acupuncture significantly reduced PLC pain; acupuncture method, treatment duration, and patient age were identified as main factors affecting efficacy | 1 | 9/11 | Investigational therapy |
| Zhang Y et al. 2023  [64] | Systematic review & meta-analysis of RCTs | Cancer patients (13 RCTs, n=1000) | Cancer pain | Massage therapy vs usual care/placebo | Massage significantly reduced cancer pain (SMD=−1.16, 95% CI [−1.39, −0.93], P<0.00001); effective in hematological malignancies, breast cancer, digestive cancers; perioperative and chemotherapy patients | 1 | 9/11 | Investigational therapy |
| Geyik Gİ et al. 2021  [68] | Randomized controlled trial | Cancer patients undergoing hematopoietic stem cell transplantation | Physical and mental symptoms during HSCT | Music therapy | Music therapy may improve physical parameters and mental parameters in HSCT patients | 2 | 9/13 | Investigational therapy |
| Köhler F et al. 2020  [69] | Systematic review & meta-analysis of RCTs/CCTs | Adult cancer patients | Psychological well‑being, quality of life, physical symptom distress | Music therapy by trained therapist | Small but significant effects: psychological well‑being (d=0.35), physical symptom distress (d=−0.26), quality of life (d=0.36); most promising during curative treatment and palliative care | 1 | 9/11 | Investigational therapy |
| Li Y et al. 2020  [70] | Systematic review & meta-analysis of RCTs | Cancer patients (19 RCTs, n=1,548) | Cancer-related pain, anxiety, depression, quality of life | Music therapy | Significantly improved overall quality of life; reduced anxiety, depression, and pain compared with standard care | 1 | 9/11 | Investigational therapy |
| Trigueros‑Murillo A et al.  2023  [71] | Overview of systematic reviews with meta‑analysis | Adults with cancer (13 SRs, 119 RCTs, >9000 participants) | Cancer‑related pain, fatigue, distress | Music‑based interventions combined with usual/standard care | Music + usual care more effective than usual care alone for pain, fatigue, distress; mixed/inconclusive for anxiety, depression, mood, QoL | 1 | 8/11 | Investigational therapy |
| Alonso Puig M et al. 2020  [72] | Quasi‑experimental pre/posttest | Pediatric cancer patients with grade 3–4 chemotherapy‑induced mucositis (n=20) | Acute pain due to severe mucositis | Electronic video games (EVGs) played on‑demand; average 2.3 h/day; continued standard PCA morphine | Significant reduction in incidental pain (7.7 to 5.4, P=0.001), resting pain (4.8 to 3.2, P=0.01), daily morphine (35.9 to 28.6 μg/kg/d, P=0.003), bolus demand (17 to 9.6, P=0.001); increased vagal tone (ANI 62.1 to 71.9, P=0.009) | 4 | 8/10 | Investigational therapy |
| Vieira C et al. 2021  [74] | Systematic review | Patients with motor dysfunction (12 studies, 512 patients) | Upper limb motor rehabilitation | Serious games (SG) intervention | 8/12 studies showed significant improvement in at least one clinical outcome; custom‑made casual games showed the best clinical outcomes | 1 | 9/11 | Investigational therapy |
| Dunn KE et al. 2024  [77] | Randomized, double‑blind, placebo‑controlled within‑subject human laboratory study | Healthy adults with limited opioid exposure (N=100) | Not a therapeutic intervention | Hydromorphone vs placebo | AG/GG genotype associated with more pleasant and fewer unpleasant effects vs AA genotype; less physiological reactivity | 2 | 11/13 | Investigational therapy |
| Salama V et al. 2024  [79] | Systematic review | Cancer patients with pain (44 studies) | Cancer‑related pain | Artificial intelligence / machine learning (AI/ML) models | AI/ML models showed good discrimination (median AUC 0.77); highest for cancer pain research (median AUC 0.86) | 1 | 9/11 | Investigational therapy |

Note: The Joanna Briggs Institute (JBI) Evidence-Based Healthcare Center standards were used for evidence grading and methodological quality assessment. The JBI evidence levels are divided into five levels: Level 1 for systematic reviews or meta-analyses (RCTs); Level 2 for randomized controlled trials; Level 3 for quasi-randomized controlled trials or cohort studies (prospective/retrospective); Level 4 for case series, case-control studies, or cross-sectional studies; and Level 5 for basic research (animal studies, in vitro experiments), case reports, or expert opinions. Level 1 represents the highest strength of evidence, and Level 5 represents the lowest. The JBI quality score is calculated item by item using specific appraisal checklists corresponding to each study type; a higher score indicates better methodological quality of the study.

*Levels of evidence (JBI Level) and quality scores are based on the Joanna Briggs Institute (JBI) Levels of Evidence and corresponding appraisal checklists for each study design, as described in the JBI Manual for Evidence Synthesis. Available online at https://jbi-global-wiki.refined.site/space/MANUAL.
